# British surname origins, population structure and health outcomes—an observational study of hospital admissions

**DOI:** 10.1038/s41598-022-05651-8

**Published:** 2022-02-09

**Authors:** Jakob Petersen, Jens Kandt, Paul A. Longley

**Affiliations:** 1 Consumer Data Research Centre (CDRC), Department of Geography, University College London (UCL), Gower Street, London, WC1E 6BT UK; 2 The Bartlett Centre for Advanced Spatial Analysis (CASA), UCL, Gower Street, London, WC1E 6BT UK

**Keywords:** Epidemiology, Socioeconomic scenarios

## Abstract

Population structure is a confounder on pathways linking genotypes to health outcomes. This study examines whether the historical, geographical origins of British surnames are associated with health outcomes today. We coded hospital admissions of over 30 million patients in England between 1999 and 2013 to their British surname origin and divided their diagnoses into 125 major disease categories (of which 94 were complete-case). A base population was constructed with patients’ first admission of any kind. Age- and sex-standardised odds ratios were calculated with logistic regression using patients with ubiquitous English surnames such as “Smith” as reference (alpha = .05; Benjamini–Hochberg false discovery rate (FDR) = .05). The results were scanned for “signals”, where a branch of related surname origins all had significantly higher or lower risk. Age- and sex-standardised admission (alpha = .05) was calculated for each signal across area deprivation and surname origin density quintiles. Signals included three branches of English surnames (disorders of teeth and jaw, fractures, upper gastrointestinal disorders). Although the signal with fractures was considered unusual overall, 2 out of the 9 origins in the branch would only be significant at a FDR > .05: OR 0.92 (95% confidence interval 0.86–0.98) and 0.70 (0.55–0.90). The risk was only different in the quintile with the highest density of that group. Differential risk remained when studied across quintiles of area deprivation. The study shows that surname origins are associated with diverse health outcomes and thus act as markers of population structure over and above area deprivation.

## Introduction

It is well established that the distribution of surnames correlates with that of genetic population structure^1–3^. In Britain, surnames have been passed down the generations for more than seven centuries^4^. When the geographical distributions of surnames are mapped with data from nineteenth century censuses, it becomes clear that many surnames can be traced back to very specific localities prior to the large-scale urbanisation and migration that characterise the people of the British Isles today^5^. Despite migration and mixing of populations—many of these surnames are still most common in the same heartlands as they were in the nineteenth century^6^. Genetic studies have found that the rarer the surname, the more likely the bearers of that surname are related^4^. This reflects that many more common surnames have multiple apparent origins, indicating different, unrelated, ancestors. Among the more widespread surnames with multiple origins are names taken from professions (e.g., Smith) or landscape features (e.g., Ford). Previous work has characterised the regionality of British surnames defining so-called isonymy regions based on the 1881 Census geography^6–8^. Isonymy regions are geographical regions whose populations bear a distinct constellation of surnames. We will refer to isonymy regions as surname origins hereinafter. One study identified direct correspondence between surname origins in 1881 and contemporary, genetic population structure^9^. Surname geographies can also reveal patterns of migration and social mobility^10^.

In theory, bearers with the same surname origin are more likely to be related genetically and culturally. How related they are and whether surname origins could be a useful marker of co-ancestry in health studies is an ongoing research concern^4^, but evidence from multiple countries suggests that present day surname bearers resident in high density heartlands are more likely to be related than bearers scattered across lower density regions, where the chance of admixture is greater.

Population structure also acts as a strong confounder which continues to limit the validity of genome-wide association studies^11^, and there is a need for methods that can systematically identify population structure and inform the sampling design of such studies.

Population structure is an outcome of ‘biosocial’ processes, i.e. entangled biological and social processes^12^. Thus biogenetic differentiation is shaped by social selection phenomena such as assortative mating, socio-cultural and geographic isolation and selective migration. Linking hospital records, for the first time, with within-nation, regional surname origins opens opportunities to capture population structure for all patients and detect variations in health﻿ while considering social characteristics such as social deprivation. The aim of the study is therefore, in the first instance, to test associations between hospital admissions and surname origins while controlling for social deprivation.

We investigate whether British surname origins can be associated with health outcomes in a large database study of hospital admissions in England today. We divide the admissions of over 30 million patients into 125 major disease categories for the study. Even though administrative hospital data are sparse on contextual data, we make provisions for this in two ways. First, we develop a dose–response relationship between health outcomes and density of different surname groups. Second, we study confounding socio-economic factors by coding patients’ residences to a national index of area deprivation.

## Methods

British surnames were identified using the ONOMAP tool, which identified clusters of non-random associations between forenames and surnames in large population datasets such as electoral registers and telephone directories from several countries^13^. The list of British surnames was first validated in a linkage with the Scottish birth register^14^ and later with the 2011 Census for England and Wales^8^. Both these validation exercises showed that the British surname classification had high sensitivity and specificity for predicting self-reported White Scottish and White British ethnicity in these data sources, respectively. Recently, this work has been taken further by linking the list of British surnames to the geographical location in census returns from the 1881 Census for England, Scotland, and Wales^7^, which has resulted in the classification of British surname origins used in this study.

The classification of British surname origins is defined using the regional concentrations of each British surname across Great Britain in 1881. They are based on the concept of ‘isonymy’, defined by “the recurrence of the same surnames in different ancestral lines in the same pedigree”^15^. This concept can be applied to measure the relatedness of zonally defined populations^2,16^, wherein zonal populations are compared pair-wise with regard to their similarity of surname compositions^9^. The resulting pair-wise estimates of degrees of isonymy are then converted to a distance measure and segmented using Ward's hierarchical clustering algorithm to identify groups of zones that have similar surname compositions. As has been previously reported^17^, there is a very high level of geographic contiguity in the cluster assignments of long-settled names, and the results of the Ward cluster analysis can be used to derive ‘isonymy regions’ delineating the probable geographical and historic origin of a surname. Each of the 88,457 surnames is assigned to an appropriate isonymy region based on this procedure^7^.

**Figure 1:**
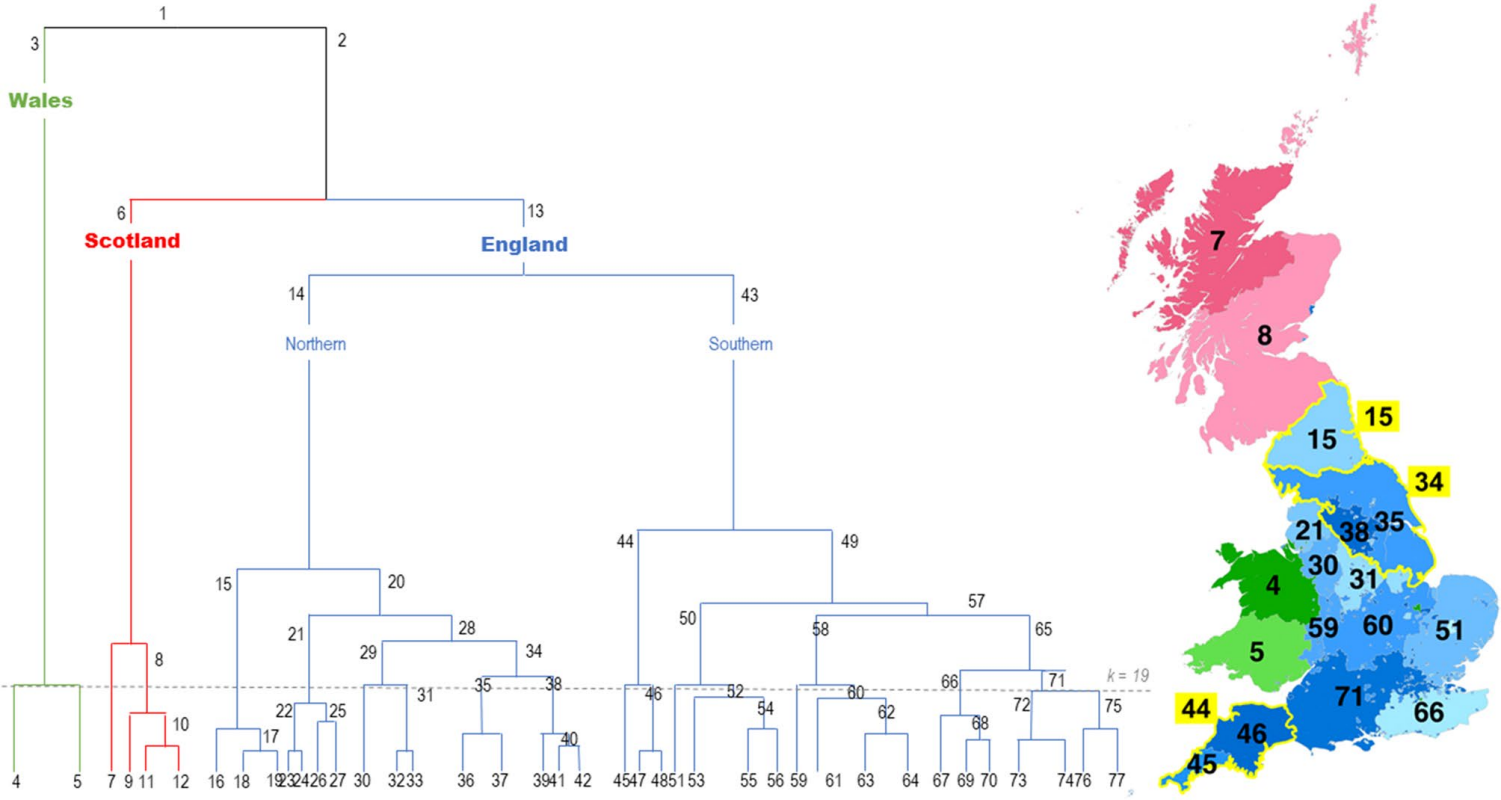
Dendrogram of hierarchical isonymy regions and the spatial extents of k = 19 regions with regions 6, 15, 34 and 44 highlighted.

We coded Hospital Episode Statistics (HES) recorded between April 1999 and March 2014 in England by the geographic origin of their surnames. The coding was carried out by NHS Digital staff in their secure environment based on our instructions in 2015. The HES Patient Demographics Service (PDS)^18^ was used to link the maiden name or first recorded surname of a patient to 1 of 74 surname origins as identified in the 1881 Census using the procedures developed by Kandt & Longley 2018 and Kandt et al. 2020 (Fig. 1)^7,8^. The PDS is continuously updated and time period when each surname was first recorded vary by patient. The full surname classification counts 77 distinct origins of which 74 were present in HES. Ethical approval was obtained from Bromley REC (Reference: 13/LO/1355) and further approval was granted by NHS Digital (HES data licence reference is DARS-NIC-28051-Q3K7L). The study was conducted in accordance with relevant guidelines and regulations. As for other non-identifiable database studies, it was not practical nor desirable to obtain consent from each patient. The study only used routinely collected, secondary data and as such involved no experimental components requiring additional protocols and approvals. The anonymised HES extracts were stored and processed under secure settings.

Diagnoses were coded using the International Classification of Diseases (ICD10) system^19^, which for analytical purposes was aggregated into 125 Clinical Classifications Software (CCS) categories^20^ (Supplementary Table S1). A denominator dataset was created with the first admission (of any kind) from each of 32,860,835 patients (72.9%) with known residence recorded between April 1999 and March 2014. In this process, 12,247,069 patients (27.2%) without surname records or non-British surnames were excluded. Only patients’ first admission for each disease category was kept. In the interest of keeping specificity and avoiding dilution of any associations, the analyses were carried out in an initial “signal detection” round followed by a second round of more detailed analyses of potential signals. Age- and sex-adjusted odds ratios for each of the 125 CCS disease categories were estimated for all 74 surname origins using logistic regression. For these analyses, patients from the dominant Cluster-13, whose origin is coterminous with England as a whole, was used as a reference population. Only 94 out of the 125 CCS categories had sufficient data for analysis, i.e. complete case. “Signals” were identified from this first round where the names making up a whole branch of related surname origins had admission odds ratios (alpha = 0.05) significantly above or below the reference population while controlling for Benjamini–Hochberg false discovery rate of 5%^21^. The rationale for defining signals in this way was that branch-level clustering suggests a lineage effect between individuals sharing surname origin and potentially also genetic and cultural roots. Age- and sex-standardised rates of admissions per 100,000 population were then calculated, weighted according to the 2013 European Standard Population^22^ of the identified signals, and broken down by origin density quintiles and area deprivation quintiles. The origin density was constructed as the percentage of patients with a given regional origin relative to all patients resident in each local authority district, separated into quintiles. Local authority was chosen as the unit of analysis because all the signal origins would have an unbroken, non-zero, distribution. A dispersion ratio for each origin aggregate was calculated as Q1 divided by Q5. Patients’ residence at the time of each admission was coded with area deprivation quintiles at neighbourhood level (MSOA11) using the Indices of Multiple Deprivation (IMD) for England 2015. The IMD is a composite index of a range of socio-economic indicators^23^.

## Results

**Figure 2:**
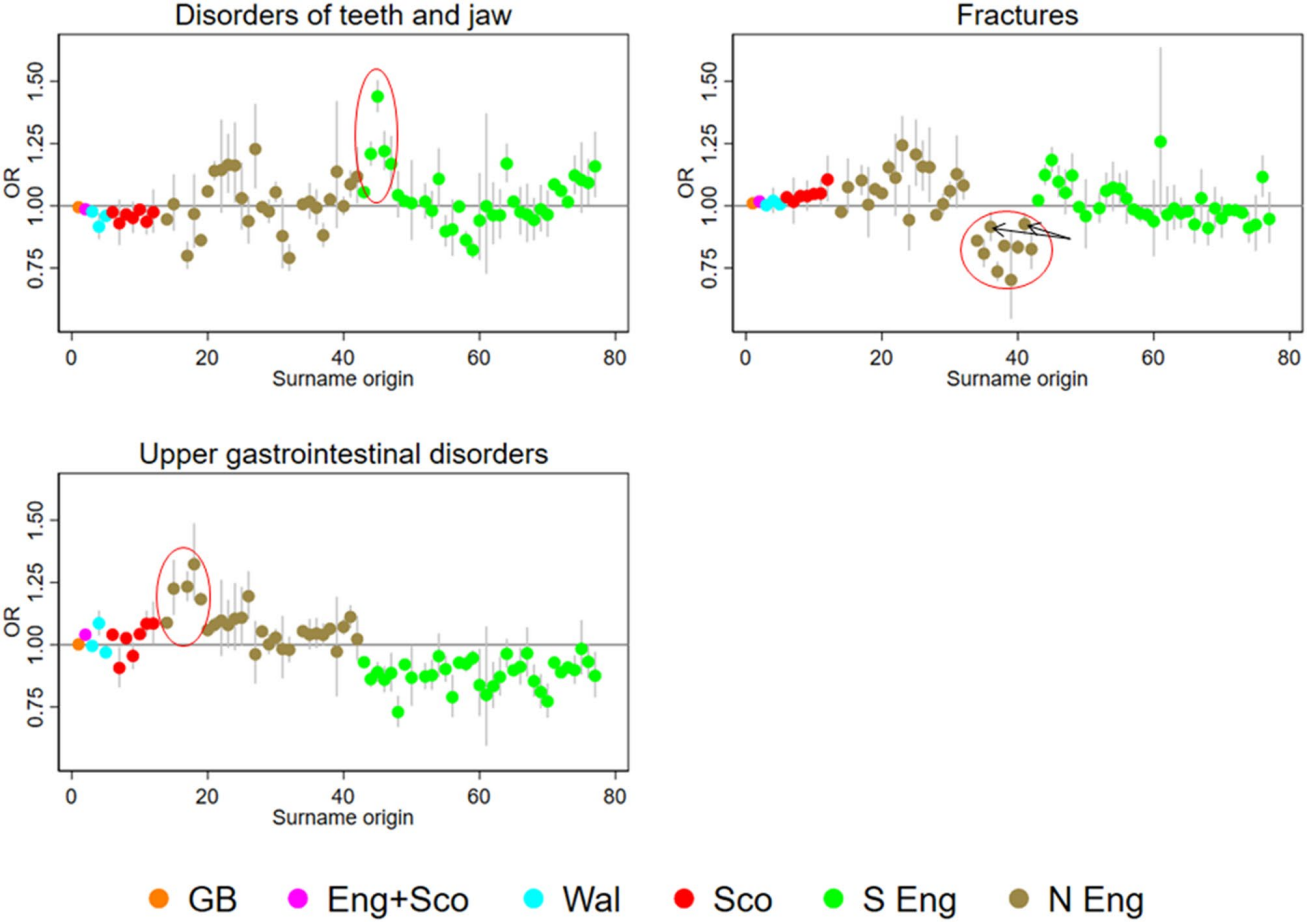
Hospital admission risk by surname origin for four disease-origin branch signals. The x-axis has the order of each surname origin in the dendrogram shown in Fig. 1 putting each “branch” next to its distal sub-branch. Arrows indicate 2 origins in the Fractures signal that would only be significant at a FDR > .05.

**Figure 3:**
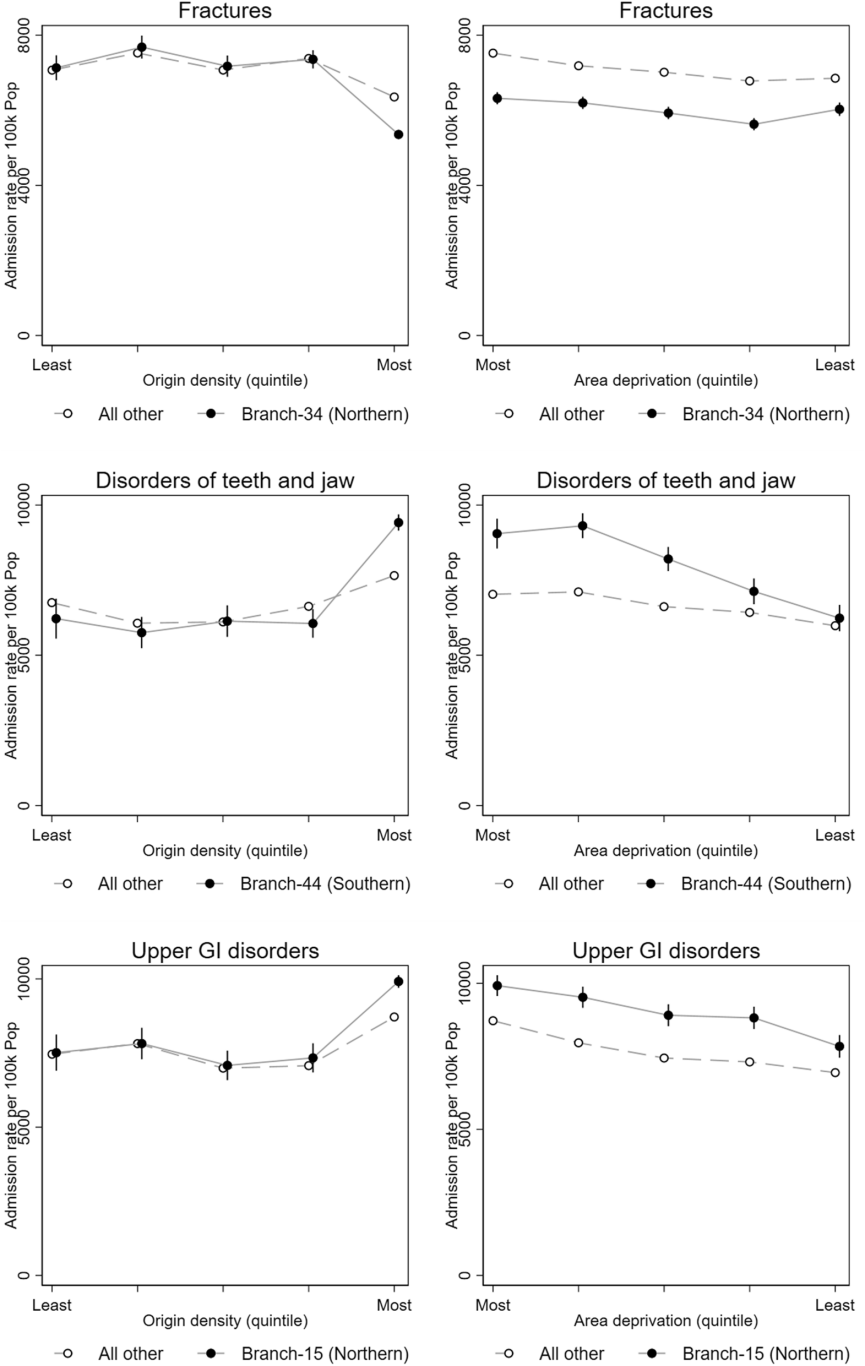
Admission rate by origin density and area deprivation quintiles.

Associations between 94 complete-case CCS disease categories and 74 different surname origins for 32,860,835 patients were studied and plotted during the screening round. The denominators for the analyses are shown in Table 1 and the numerators (case patient numbers) for each signal in Table 2. The case numbers varied from 6,665 teeth and jaw disorder patients with Branch-44 (Southern) surnames to 25,218 fracture patients with Branch-34 surnames (Northern) (Table 2). The dispersion ratio (Q1/Q5) varied from 0.09–0.10 (Table 1). The hospitalisation odds ratios for the signals are shown in Fig. 2. Three branches with English surnames were identified as signals with all containing clusters either significantly above or below the reference group while controlling for a false discovery rate of 5%. Although the signal with fractures was considered unusual overall, 2 out of the 9 origins in the branch would only be significant at a FDR > 0.05: OR 0.92 (95% confidence interval 0.86–0.98) and 0.70 (0.55–0.90). The age- and sex-standardised admission rates (and 95% confidence intervals) for each signal disease were broken down by origin density and area deprivation quintile (Fig. 3). The risk was only different in the quintile with the highest density of that group. Differential risk remained when studied across quintiles of area deprivation.

**Table 1:** Patient denominator characteristics by signal set surname origins, reference surname origin (Cluster-13), and total

	Branch-44 surnames (Southern)	Branch-34 surnames (Northern)	Branch-15 surnames (Northern)	Reference Cluster-13 surnames (England)	Total
**Sex**					
Male	48,535	200,665	59,059	5,561,932	15,106,398
Female	56,770	234,030	68,495	6,526,510	17,754,437
**Age (years)**					
< 20	23,561	96,366	29,317	2,731,390	7,424,172
20–39	23,687	99,405	29,708	2,817,877	7,722,194
40–59	23,911	99,843	30,390	2,770,088	7,536,888
60–79	24,854	102,800	29,160	2,786,014	7,525,759
80 +	9292	36,281	8979	983,073	2,651,822
**Origin density (quintile)**^**a**^					
1. Least common	6403	27,114	8030	–	–
2	10,148	33,269	11,089	–	–
3	10,251	36,530	11,505	–	–
4	12,907	51,626	12,203	–	–
5. Most common	65,596	286,156	84,727	–	–
Dispersion ratio (Q1/Q5)	(0.10)	(0.09)	(0.09)	–	–
**Area deprivation (quintile)**					
1. Most deprived	16,966	97,508	32,676	2,457,262	6,516,508
2	26,267	91,076	28,622	2,394,096	6,478,882
3	25,836	86,115	24,266	2,435,752	6,659,466
4	19,773	86,932	22,675	2,453,281	6,725,002
5. Least deprived	16,463	73,064	19,315	2,348,051	6,480,977
**Total**	105,305	434,695	127,551	12,088,442	32,860,835

First admission of any kind in patients with British surnames in Hospital Episode Statistics (HES) in England between 1999 and 2013.

^a^Origin density is specific to the signal and not shown for reference population and totals

**Table 2:** Patient characteristics by signal (disease category-surname origin) and totals in number of patients.

	Disorders of teeth and jaw		Fractures		Upper gastrointestinal disorders	
	Branch-44 surnames (Southern)	All other surnames	Branch-34 surnames (Northern)	All other surnames	Branch-15 surnames (Northern)	All other surnames
**Sex**						
Male	2,945	756,995	11,782	1,030,431	5,542	1,203,775
Female	3,720	958,421	13,436	1,150,123	5,886	1,282,559
**Age (years)**						
< 20	2,169	584,483	4,700	430,858	441	101,273
20–39	2,484	663,029	4,295	364,617	1,541	322,634
40–59	1,244	299,598	3,977	335,095	3,932	827,616
60–79	653	142,602	5,629	484,692	4,545	981,616
80 +	115	25,704	6,617	565,292	969	253,729
**Origin density (quintile)**^**a**^						
1. Least common	336	432,728	1,783	443,771	584	503,411
2.	470	374,574	2,425	450,592	834	558,909
3.	531	266,907	2,462	399,554	781	440,546
4.	622	275,937	3,533	459,611	857	391,981
5. Most common	4,706	365,270	15,015	427,026	8,372	591,487
**Area deprivation (quintile)**						
1. Most deprived	1,276	384,103	5,881	444,551	2,969	512,185
2.	1,946	371,057	5,421	433,988	2,665	494,342
3.	1,602	343,270	4,979	444,085	2,163	499,157
4.	1,.067	327,171	4,626	434,238	2,061	508,585
5. Least deprived	774	289,815	4,311	423,692	1,570	472,065
**Total**	6,665	1,715,416	25,218	2,180,554	11,428	2,486,334

First admission per patient per disease group by British surname origin status for each signal in Hospital Episode Statistics (HES) in England between 1999 and 2013.

## Discussion

The main objective of this research is to examine whether the origin of patients’ surnames in 1881 act as plausible markers of population structure that are associated with health outcomes in a large population-wide dataset today. Given that most origin groups are relatively sparsely powered, we decided to screen the results for signals where an entire branch of related origins had significantly higher or lower risk than the reference population of pan-English surname bearers.

We found three such signals. All the signals were associated with greater (dis-)advantage in the high-density regions compared to the low-density regions.

The analysis of area deprivation showed that disadvantage increased with area deprivation relative to patients with all other surname origin for teeth and jaw disorders. For fractures and upper gastrointestinal disorders, the relative (dis-)advantage changed little across deprivation quintiles.

The results suggest that it is possible to detect differential health outcomes linked to the ancestral “heartland” and that the effects persist even when considering variation across different levels of area deprivation.

From a genetic perspective, it could be hypothesised that the higher risk was caused by inbreeding depression or genetic drift. The fact that the identified signals are not headline diseases with a big impact on patients’ lives or healthcare budgets, could arise because they are exactly marginal diseases under low selection pressure. Inbreeding depression is classically associated with consanguineous relationships and monogenic diseases, but nascent research suggest that it can also be associated with complex diseases and other complex traits even in population-based samples^24,25^.

From a social science perspective, an alternative explanation could be that the surname classification likely capture a wide range of variables associated with “nurture”, which again may differ between heartlands and the “host” regions.

As posited by dual inheritance theory, genetic and cultural roots are intertwined^26^. While genetic inheritance can only be vertical, cultural inheritance can both be horizontal and vertical. Cultural practices can thus be passed down vertically from forbears or quickly adopted from peers horizontally. Wealth accumulated in families can be an example of vertical, cultural transmission. Which set of factors, vertical or horizontal, genetic or cultural, are dominant will depend on the particular aetiology of the health condition in question.

A motivation for further studies would either be to contextualise surname origins as a new geography or to study the fine-scale population structure where it has implications for the design of genetic studies^27^. The former approach should refine the regional geography used here in order to accommodate the likely effects of migration of family groups prior to the collection of censuses that have been digitally encoded to date.

A few limitations should be acknowledged. HES was created for administrative and billing purposes, but the data have been validated for research^28,29^. It cannot be ruled out that there could be sub-national coding differences especially when studying the entire range of diagnoses as in this case. The purpose of a medical classification such as CCS is to break down the analyses into meaningful categories. As with any categorisation, important variation may be lost and vary depending on the system deployed.

The base population was created from HES itself, i.e., as patients with any diagnosis by surname origin. In this way it was possible to study individual surname origin groups against the dominant group with ubiquitous English surnames. The results may be biased if the patient populations are not generalisable to the populations with the same surname origin and we were not able to validate this aspect of the analyses due to the lack of external reference population data.

To avoid distorting representations of health conditions associated with multiple admissions, we only used the first admission for each condition and for each patient in the base population. With data collection spanning fifteen years, there could be a bias towards exposures in patients’ younger years. We assumed that any potential biases from this source would cancel each other out although there could be residual net bias if the age of onset varied markedly between the surname origin and the reference population. In addition, HES does not contain identifiers for households, which for our analyses may mean that there could be residual clustering at household level, e.g., poisoning incidents involving multiple household members with identical surname origin.

A patient may change surname, e.g., following marriage, and end up being coded to a different surname origin than the one at birth. This would make the dataset noisier, but we reduced this by only using the maiden or first-recorded surname for each patient. In addition, local intermarriage is still common and thus it can be expected that the newly adopted surname may belong to the same or a closely related surname origin^30^.

Retirement migration may mean that exposures causing some health problems are systematically attributed to retirement regions. This is a limitation, although in this study it would only apply if the retiree moves into a different area deprivation quintile since all other variables would remain the same.

The area deprivation index used in this study is admittedly a very reductionist measure conflating diverse conditions and experiences that can lead to poorer health outcomes. Further research should therefore include more detailed data on individual socio-economic factors.

We studied 94 different disease categories across 74 surname origins. Especially for the first round, this constitutes a multiple comparison problem. We decided to focus on signals where groups of surname origins differed from the reference population on the basis that “lineage” would provide a higher degree of plausibility and crucially reduce the number of simultaneous analyses. Furthermore, we applied a 5% Benjamini–Hochberg false discovery rate to the p-values in the signal-detection round to address this problem^21^.

## Conclusion

The study shows that surname origins are associated with diverse health outcomes and may thus act as combined markers of biosocial population structure over and above area deprivation. If related surname origins were studied—assuming either genetic or cultural lineage effect—then again it was possible to find correlates between certain health outcomes in the areas with the highest density of the surname origin aggregate. The fact that correlates were only present in the highest density quintile suggests that ancestral heartlands vary in some aspect from the rest of the country. Hypothetically this pattern could be explained by a combination of factors related to both nature and nurture, in part depending on the nature of the health outcome itself. The results may thus inform more detailed investigations untangling biological and social factors in health.

## Supplementary Information

Supplementary Information.
